# Dihydromyricetin Acts as a Potential Redox Balance Mediator in Cancer Chemoprevention

**DOI:** 10.1155/2021/6692579

**Published:** 2021-03-11

**Authors:** Liang Chen, Meng Shi, Chenghao Lv, Ying Song, Yuanjie Wu, Suifei Liu, Zhibing Zheng, Xiangyang Lu, Si Qin

**Affiliations:** ^1^College of Bioscience and Biotechnology, Hunan Agricultural University, Changsha 410128, China; ^2^College of Food Science and Technology, Hunan Agricultural University, Changsha 410128, China; ^3^Hunan Tea Group Co., Ltd., Changsha 410128, China; ^4^Jiangxi Agricultural Engineering College, Yichun 331200, China

## Abstract

Dihydromyricetin (DHM) is a flavonoid extracted from the leaves and stems of the edible plant *Ampelopsis grossedentata* that has been used for Chinese Traditional Medicine. It has attracted considerable attention from consumers due to its beneficial properties including anticancer, antioxidative, and anti-inflammatory activities. Continuous oxidative stress caused by intracellular redox imbalance can lead to chronic inflammation, which is intimately associated with the initiation, promotion, and progression of cancer. DHM is considered a potential redox regulator for chronic disease prevention, and its biological activities are abundantly evaluated by using diverse cell and animal models. However, clinical investigations are still scanty. This review summarizes the current potential chemopreventive effects of DHM, including its properties such as anticancer, antioxidative, and anti-inflammatory activities, and further discusses the underlying molecular mechanisms of DHM in cancer chemoprevention by targeting redox balance and influencing the gut microbiota.

## 1. Introduction

**Figure 1:**
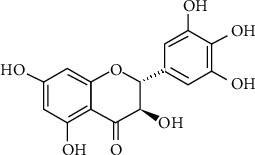
Chemical structure of dihydromyricetin (DHM).

*Ampelopsis grossedentata* (*A. grossedentata*) is a medicinal and edible plant widely used in China as a Traditional Chinese Medicine for the treatment of cough, fever, vomiting, hepatitis, colds, chronic nephritis, polyorexia, and sore throat. Tender stems and leaves of *A. grossedentata* are commonly consumed as vine tea in China for centuries due to its health benefiting effects. Dihydromyricetin (3,5,7,3′,4′,5′-hexahydroxy-2,3-dihydroflavonol (DHM)) (Figure 1), also known as ampelopsin, is a major flavonoid extracted from the leaves and stems of *A. grossedentata.* The content of DHM in *A. grossedentata* ranges from 30% to 40% (dry weight), which is considered to have the highest flavonoid content in natural plants [1]. Several scientific investigations reported that DHM possesses various biological activities such as anti-inflammatory [2, 3], antioxidative [4, 5], anticancer [6, 7], antidiabetic [8–10], antiatherosclerosis [11, 12], and cardioprotective effects [13, 14]. The biological properties and the underlying mechanisms of DHM were investigated mostly by *in vitro* cell cultures and *in vivo* animal models. In addition, DHM was reported as toxicologically safe and could effectively reverse multidrug resistance [15–19]. Hence, DHM is a promising bioactive compound for developing healthy/functional foods.

Despite these health-promoting effects, DHM has very poor water-solubility and aqueous stability. The solubility characteristics of DHM in cold water, hot water, and ethanol were 0.2-0.32 mg/mL at 25°C, 20 mg/mL at 80°C, and 170 mg/mL at 25°C, respectively [20]. DHM is more stable in acidic conditions (pH range of 1.0-5.0) than in an alkaline environment. Under alkaline conditions, especially at a pH range from 6.0 to 8.0, DHM was prone to oxidation and degraded dramatically [21]. Similarly, the stability of DHM is affected by the pH rather than the digestive enzymes including pepsin and pancreatin under the *in vitro* digestion system [21]. At a concentration of 20 *μ*g/mL, DHM was stable at room temperature after 12 h and at -20°C for 10 d, but only 45.42% was retained after 3 h in simulated intestinal fluid at 37°C [21]. The plasma DHM concentration reached maximum (159 *μ*g/L) at 1.5 h postadministration when DHM powder was given at a dosage of 115 mg/kg body weight in rabbits, indicating a low bioavailability of DHM [18]. Efflux transporters, multidrug resistance protein 2, and breast cancer resistance protein also played an important role in DHM uptake and transport processes [22]. A research group had investigated the distribution, excretion, and metabolic profile of DHM and found that most unconverted DHM forms were excreted in feces [23]. Eight metabolites of DHM in urine and feces were found to be linked with reduction, methylation, dehydroxylation, glucuronidation, and sulfation metabolic pathways [23]. These problems together resulted in its low membrane permeability (*P*_eff_ = (1.84 ± 0.37) × 10^−6^ cm/s) and compromised bioavailability [18]. However, the amount of DHM from the daily intake of *Ampelopsis grossedentata* that can possibly exert its bioactivity had been investigated in clinical trials. For instance, the administration of DHM at 970 mg/day was reported to effectively ameliorate the glycemic control in type 2 diabetes mellitus in a previous study [18]. In a double-blind clinical trial, daily uptake of 600 mg of DHM exerted anti-inflammatory effects on patients with nonalcoholic fatty liver disease [18].

Through identification and quantification methods, the transport mechanisms and protective effects of DHM in metabolic diseases have recently been reviewed [18, 24]; however, the anti-inflammatory, antioxidative, and anticancer effects and their underlying molecular mechanisms have not been fully documented. This study is aimed at giving an overview of the anti-inflammatory, antioxidative, and anticancer effects of DHM, as well as recent findings regarding its underlying molecular mechanisms including redox balance and the role of gut microbiota.

## 2. Dihydromyricetin Exerts Its Chemopreventive Potential against Cancer

Cancer is a public health problem and the leading cause of morbidity and mortality worldwide. The redox imbalance involving persistent chronic inflammation and reduced antioxidant capacity are the critical pathological causes of cancer. Presently, chemoprevention is a major approach to prevent the growth of cancer cells. However, high cost and side effects associated with chemotherapy have prompted scientists to search for safe alternative natural compounds for cancer therapy [25]. Flavonoids are plant phytochemicals, and several epidemiological studies have reported that flavonoid intake may prevent a variety of cancers such as lung, breast, prostate, pancreas, and colon cancers [26].

DHM, a flavonoid from the edible plant *Ampelopsis grossedentata*, exhibited anticancer activity against a variety of cancer cells in various cultured cancer cells and animal models transplanted with cancer cells, as shown in Table 1. The most widely used cell lines for the determination of anticancer effects of DHM were HepG2 and SK-Hep-1 (human hepatocellular carcinoma), MCF-7 and MDA-MB-231 (human breast cancer), PC-3 (human prostate cancer), A549 and H1975 (human non-small-cell lung cancer), U251 and A172 (human glioma), SKOV3 (human ovarian cancer), SGC7901 and SGC7901/5-FU (human gastric carcinoma), and JAR (human choriocarcinoma) [27–39]. Different cancer cell lines were used by various researchers as each cell line has a different origin, tumor characteristics, and signaling pathways. In addition, animals such as mice and rats with transplanted cancer cells have also been used as *in vivo* models for investigation of antitumor activity of DHM [31, 37, 40, 41].

DHM effectively showed anticancer activity in a variety of cancers such as breast cancer, hepatocellular carcinoma, melanoma, ovarian cancer, lung cancer, cervical carcinoma, glioma, and osteosarcoma [32, 33, 42–46]. Among the treatments for different cancer cells, DHM has a broad dosage from 1 to 1000 *μ*M with a duration from 6 to 72 h, presenting cell proliferation inhibition and apoptosis-inducing effects (Table 1). The concentration of DHM used in various studies shifts dramatically, which might be due to the differences in cell lines, DHM purity, and cell treatment conditions. The functional mechanisms and major pathways are also listed in Table 1. DHM inhibited the proliferation of HepG2 cells via G2/M phase cell cycle arrest through the Chk1/Chk2/Cdc25C signaling pathway; induced the apoptosis of HepG2 cells that target ROS-related, Akt/Bad, ERK1/2, AMPK, and PI3K/PDK1/Akt signaling pathways; enhanced the levels of DR4, DR5, Bax, Bad, and caspase 3; and reduced the expression of Bcl-2 protein and mTOR [30, 32, 47, 48]. The suppressing effect of DHM on the MDA-MB-231 breast cancer cell line was reported through ROS generation, ER stress pathway, and inhibition of mTOR [28, 31]. DHM treatment dose-dependently inhibited the growth of HeLa cells by inducing apoptosis through activation of caspases 9 and 3 and increasing the ratio of Bax protein to Bcl-2 [44]. In A549 human adenocarcinoma lung epithelial cells, DHM decreased XIAP and survivin expression levels and cleaved poly(ADP-ribose) polymerase. DHM stimulated apoptosis via a p53-mediated pathway in ovarian cancer cells A2780 and SKOV3 [46]. DHM was also reported to inhibit human melanoma SK-MEL-28 cells by inducing apoptosis; arresting cell cycle at the G1/S phases; increasing the production of p53 and p21 proteins; enhancing the expression levels of Bax proteins; and decreasing the protein levels of IKK-*α*, NF-*κ*B (p65), and P-p38 [49]. In addition, DHM suppressed the glioma cell growth through enhancing apoptosis; arresting the cell cycle at the G1 and S phases; and activating caspase 8, caspase 9, and caspase 3 [37]. DHM exhibited anticancer activity in osteosarcoma cells through G2-M cell cycle arrest, DNA damage prevention, stimulation of the ATM-CHK2-H2AX signaling pathways, and enhancing p21 expression [50]. Zuo et al. reported that DHM suppressed the growth of human choriocarcinoma JAR cells by inducing cell cycle arrest and reducing the expression levels of cyclin A1, cyclin D1, SMAD3, and SMAD4. DHM function on other cancer cells is likely to share similar pathways. However, few studies have investigated the cytotoxic effects of DHM on normal cells. The anticancer effects should selectively inhibit the growth of the cancerous cells without damaging the normal cells. The lack of cytotoxic data on normal cells could potentially limit the use of DHM as an anticancer agent.

Several studies evaluated the anticancer effects of DHM in combination with anticancer drugs in order to overcome the drug resistance of cancerous cells. DHM in combination with nedaplatin (anticancer drug) showed a synergistic effect on the inhibition of the growth of hepatocellular carcinoma cells SMMC7721 and QGY7701, and induced apoptosis through the activation of the p53/Bcl-2 signaling pathways [51]. Also, DHM in combination with erlotinib significantly induced the caspase-dependent cell death in NSCLC due to a synergistic effect [43]. More importantly, interactions between DHM and other drugs and their toxicological properties need to be substantially evaluated before use as anticancer drugs since DHM has the potential to show synergy effects with drugs.

Apart from cultured cancer cell lines, many researchers determined the antitumor potential of DHM in various animal models bearing transplanted cancer cells. PC-3 tumor growth was significantly reduced by 49.2% through the administration of DHM at 300 mg/kg BW in mice [27]. It was found that tumor size was significantly reduced in mice treated with DHM compared to the controls in athymic mice xenografted with MDA-MB-231 cells [31], in a nude mice xenograft model bearing the human osteosarcoma cell line U2OS/MTX, in a mice xenograft model bearing the human osteosarcoma cell line U2OS/MTX [50], and in xenograft BALB/c-nu mice transplanted with the human glioma cell line U251. Though there are some reports on the antitumor effects of DHM, there are some limitations that could hinder the advancement of DHM as an anticancer agent for human use. On the one hand, the molecular mechanism and major pathways still remain unclear. It is important to reveal the mechanism with consistency among cell models, animal models, and clinical studies. Therefore, more research is needed in animals and humans to generate reliable and consistent scientific evidence regarding the anticancer effects of DHM.

## 3. Antioxidant Capacity Is the Main Reason for the Anticancer Property of DHM

Aerobic cellular respiration generates free radicals and reactive oxygen species (ROS). The in-built antioxidant defense system protects the body from the harmful effects of free radicals. The imbalance between free radicals and the antioxidant defense system results in oxidative stress. The free radicals and ROS contain unpaired electrons in the outer shell, resulting in their instability. These unstable free radicals are highly reactive; attract electrons from other molecules; and cause oxidative damage to proteins, lipids, carbohydrates, and nucleic acids [52]. The oxidative damage inflicted upon macromolecules results in oxidative stress, which has been found to be highly associated with cancer [53].

Plant-derived flavonoids have been shown to inhibit free radicals and oxidative stress [54]. Recently, there have been many studies reporting on the antioxidant capacity of DHM. The evaluation methods, antioxidant properties, and mechanisms of DHM are shown in Table 2. Several *in vitro*, cell culture, and *in vivo* (animals) models are commonly used for the determination of the antioxidant activity of DHM, of which the most commonly used are the *in vitro* methods including free radical scavenging methods such as DPPH, ABTS, oxygen radical absorption capacity (ORAC), H_2_O_2_ radical scavenging power, and Fe^2+^ chelating method and ferric reducing antioxidant power (FRAP) [5, 55–58]. Several studies documented the *in vitro* free radical scavenging activity of DHM. The IC_50_ values measured by DPPH, ABTS, H_2_O_2_, and O_2_ radicals were 3.24-22.6, 3.1-5.32, 7.95, and 7.79 *μ*g/mL, respectively (Table 2) [5, 17, 59, 60].

DHM has been shown to protect oxidative stress in various cell culture models with a concentration below 1000 *μ*M, as shown in Table 2. Cell lines such as human hepatoma cells (HepG2), human umbilical vein endothelial cells (HUVECs), human colon cancer (Colo-205) cells, porcine kidney epithelial cells (PK-15), PC12 cells, murine macrophage (RAW264.7) cells, glomerular mesangial cells (MCs), and HEI-OC1 auditory cells have been successfully used to determine the protective effects of DHM in oxidative stress, and oxidative stress is generally created in cell lines by using H_2_O_2_ free radicals, LPS, methylglyoxal, and sodium nitroprusside [4, 41, 47, 55, 61–67].

Oxidative stress-induced mice, rat, chicken, and piglet models have also been used by many researchers to investigate the protective role of DHM in oxidative stress. The dose used in animal studies was 25 to 250 mg/kg BW, and the duration was between 2 and 3 months. In animal studies, superoxide dismutase (SOD), glutathione peroxidase (GSH-Px), glutathione (*GSH*), and malondialdehyde (MDA) are commonly measured oxidative stress parameters to estimate the antioxidant capacity of DHM [13, 68–70]. The major antioxidant effects of DHM through the Nrf2/HO-1 pathway, the Nrf2/Keap1 pathway, and the ERK and Akt pathways [5, 62, 66, 71], with an increase of DHM, significantly decreased the ROS levels in human umbilical vein endothelial cells (HUVECs) [71]. DHM exhibited an antioxidative power by activating superoxide dismutase (SOD) and the Nrf2/HO-1 signaling pathway or the Nrf2/Keap1 pathway [5, 64]. Recently, Dong et al. found that DHM exhibited antioxidative effects through the inhibition of intracellular ROS production and expression levels of ROS producing the enzymes NADPH oxidase 2 (NOX2) and NOX4, the suppression of MDA levels, the enhancement of SOD, and the activation of the Nrf2/HO-1 signaling pathway [66]. In another study, Zhang et al. determined the protective effects of DHM on HUVECs against sodium nitroprusside- (SNP-) induced oxidative damage and reported that DHM reduced ROS production and MDA levels, and increased SOD activity by activating the PI3K/Akt/FoxO3a signaling pathways in HUVECs [65]. DHM was also reported to inhibit the oxidative stress in HEI-OC1 auditory cells through the suppression of ROS accumulation [67]. DHM inhibited the activity of phase I enzymes, including cytochrome P450 (CYP), and phase II enzymes, including sulfotransferases (SULTs) and N/O-acetyltransferases (NAT1 and NAT2) [72, 73]. Oxidative stress has been involved in several neurodegenerative diseases such as Parkinson's disease, Alzheimer's disease (AD), and Huntington's disease [62]. Several researchers investigated the neuroprotective effect of DHM in oxidative stress-induced PC12 neuronal-like cells. They also investigated the neuroprotective role of DHM in neuronal-like PC12 cells against H_2_O_2_-induced oxidative stress and reported that DHM treatment at 1, 5, and 15 mg/mL for 1 h inhibited the formation of ROS and increased the cellular antioxidant defense through activation of the ERK and Akt signaling pathways in PC12 cells [62]. Jiang et al. found that DHM reduced the oxidative stress in PC12 cells by inhibiting the intracellular ROS production and by modulating the AMPK/GLUT4 signaling pathways [63].

The oxidative stress protection capacity of DHM was also evaluated in several animal models. DHM showed the highest radical scavenging activity (42.26%) of serum at 4 h after DHM administration in rats [57]. Li et al. induced oxidative stress in the brains of ICR mice by sleep deprivation and found that DHM administration significantly reduced oxidative stress by increasing SOD activity and reducing the MDA level in the hippocampus of sleep-deprived mice [69]. Similarly, for streptozotocin-induced or transverse aortic constriction surgery-induced oxidative stress in mice as well as high-fat diet-induced oxidative stress in rats, DHM decreased MDA and increased SOD, GSH, and GSH-Px [13, 74, 75]. When induced by LPS to cause oxidative stress, DHM increased total antioxidant capacity and reduced the MDA levels in piglets, and increased SOD and GSH-Px activity and GSH in chicken plasma and ileum [55, 70]. The scientific evidence from *in vitro*, cell culture, and animal studies clearly indicate that DHM could prevent the free radicals, oxidative stress, and related markers. However, scientific data related to the antioxidant capacity of DHM in humans is scanty. Therefore, more clinical investigations are needed to improve the therapeutic applications of DHM as a natural antioxidant.

## 4. Anti-Inflammatory Capacity Is Fundamental and Is the Immediate Reason for Its Anticancer Efficacy

Inflammation is a complex and normal response of the immune system to external stimuli such as pathogens, toxins, chemical agents, infection, and tissue injury. When inflammatory cells (e.g., macrophages) are activated by stimuli (e.g., LPS and IFN-*γ*), inflammatory mediators such as IL-1*β*, IL-6, TNF-*α*, NO, and PGE2 are excessively produced through the activation of common inflammatory signaling pathways such as the NF-*κ*B, MAPK, and JAK-STAT pathways [76]. The overproduction of inflammatory mediators (IL-1*β*, IL-6, TNF-*α*, NO, and PGE2) has been associated with several diseases such as diabetes, cancer, asthma, metabolic syndrome, arthritis, cardiovascular diseases, and inflammatory bowel diseases [77]. Recently, there has been growing interest in nutraceuticals and functional foods derived from plant sources. Flavonoids are polyphenolic compounds largely present in vegetables, fruits, legumes, and tea. Flavonoids such as quercetin, cyanidin, luteolin, anthocyanidin, catechin, and epicatechin have shown to contain anti-inflammatory properties [78]. DHM has been extensively studied by many researchers for its anti-inflammatory activities using various cell cultures, animal models, and human studies. Several researchers used different inflammatory models (neuroinflammation, arthritis inflammation, and lung inflammation) to investigate the anti-inflammatory potential of DHM.  Table 3 shows the various models used and the molecular mechanisms of the anti-inflammatory property of DHM.

The most widely used model for the investigation of the anti-inflammatory activity of plant-derived compounds is the macrophage that is stimulated by LPS. Macrophages play an important role in inflammation. The murine RAW264.7 macrophage cell line is the most commonly used cell culture model for the determination of the anti-inflammatory activity of food-derived compounds. Macrophages stimulated by the Toll-like receptor ligand LPS produce various inflammatory markers such as TNF-*α*, IL-6, IL-1*β*, NO, transcription factor NF-*κ*B, and prostaglandin-E2 that regulate the inflammatory responses. Numerous studies reported that DHM showed anti-inflammatory activity through different molecular mechanisms such as suppression of proinflammatory cytokines (IL-1*β*, IL-6, IL-8, and TNF-*α*), activation of the production of an anti-inflammatory cytokine (IL-10), inhibition of MAPKs, suppression of the production of prostaglandins and nitric oxide, and inhibition of the transcription factor NF-*κ*B [3, 4, 79, 80].

The inflammatory response of the brain or the spinal cord is known as neuroinflammation, and it has an important role in the development of depression by producing cytokines, chemokines, and ROS [26]. Microglial cells are macrophages in the central nervous system and are commonly used as a model to investigate the protective effects of DHM in neuroinflammation. Several researchers evaluated the anti-inflammatory capacity of DHM in neuroinflammation using microglial cells and mice models. Weng et al. investigated the neuroinflammation protection capacity of DHM using murine BV-2 microglial cells activated by LPS and reported that DHM inhibited neuroinflammation by suppressing the I*κ*B/NF-*κ*B inflammation pathway as well as decreasing STAT3 nuclear translocation and the phosphorylation levels of JAK2-STAT3. Additionally, the authors demonstrated that DHM treatment significantly inhibited the production of inflammatory mediators IL-1*β*, IL-6, TNF-*α*, nitric oxide (NO), prostaglandin E2 (PGE2), and the enzymes inducible nitric oxide synthase (iNOS) and cyclooxygenase-2 (COX-2) in LPS-induced microglial cells. DHM treatment at 10 and 20 mg/kg/day for 3 days showed an antidepressant-like activity by significantly inhibiting TNF-*α* and IL-6 gene expressions and protein levels in a mice model of LPS-induced neuroinflammation [81]. DHM at 1 mg/kg BW for 4 weeks significantly inhibited the neuroinflammation of APP/PS1 double transgenic mice by decreasing IL-1*β* via reduction of the activation of NLRP3 inflammasomes [82]. In a lead- (Pb-) induced inflammation model in mice, Pb combined with DHM administration at a dose of 125 and 250 mg/kg/BW significantly inhibited TNF-*α* and IL-1*β* and the nuclear translocation of NF-*κ*B p65 via regulating the AMPK, A*β*, TLR4, MyD88, p38, and GSK-3*β* signaling pathways. DHM exhibited an anti-inflammatory effect on LPS-induced BV-2 microglial cells by suppressing the proinflammatory markers IL-6, IL-1*β*, TNF-*α*, iNOS, and COX-2 through reducing the activation of the TRL4/NF-*κ*B signaling pathway. These studies proved that DHM possess neuroinflammation protection activity through the inhibition of inflammatory mediators.

The anti-inflammatory effects of DHM were investigated with the administration of a DHM dosage from 0.025 to 500 mg/kg BW within 2 weeks to 14 weeks. Xu et al. studied the anti-inflammatory capacity of DHM in an ovalbumin- (OVA-) induced mice C57BL/6 model of asthma and demonstrated that DHM treatment significantly decreased the levels of IL-4, IL-5, and IL-13 in the bronchoalveolar lavage fluid compared to the control group [83]. DHM treatment significantly suppressed IL-1*β* and IL-18 production by inhibiting the NLRP3 inflammasome through the activation of the SIRT1 signaling pathway in a doxorubicin- (DOX-) induced rat model and in DOX-treated H9C2 cells [84]. DHM was found to reduce the inflammation in CIA rats by attenuating IL-1*β* production through the suppression of the NF-*κ*B signaling pathway [85]. Chu et al. reported that DHM significantly reduced the inflammation in a rat model of rheumatoid arthritis by inhibiting the levels of inflammatory mediators IL-1*β*, IL-6, TNF-*α*, and COX-2 via activating the Nrf2 pathway [86]. Chang et al. investigated the protective role of DHM in ileum inflammation, induced by LPS, in chickens and found that DHM treatment decreased IL-1*β* and IL-18 expression through the inhibition of the TLR4/NF-*κ*B signaling pathways. DHM significantly decreased the proinflammatory cytokines TNF-*α*, IL-1*β*, and IL-6 and the COX-2 gene expressions and increased the production of IL-10 in the liver of piglets injected with LPS [21]. Additionally, the authors demonstrated that DHM supplementation in LPS-treated piglets decreased the activation of AKT and STAT3 phosphorylation and reduced the DNA-binding activity of NF-*κ*B [21].

More importantly, the anti-inflammatory activity of DHM was also determined in humans. Chen et al. conducted a randomized double-blind controlled clinical trial with sixty adult nonalcoholic fatty liver disease patients, and the DHM was administered (150 mg capsules) twice daily for 12 weeks [87]. The authors found that DHM exhibited an anti-inflammatory activity in humans by decreasing the serum levels of TNF-*α*, cytokeratin-18 fragment, and fibroblast growth factor 21.

Although sufficient evidence is available from cell culture and animal experiments, the results from clinical studies are meager. Therefore, it is suggested that more studies are needed in humans to further confirm the anti-inflammatory activity of DHM. More results from human studies would provide strong scientific evidence for using DHM as a therapeutic agent to treat inflammation and its related diseases.

## 5. Gut Microbiota Is a Potential Interface for the Regulation of Redox Balance for Cancer Prevention

The interface of gut microbiota is very important for cancer chemoprevention of DHM, and the interaction between DHM and gut microbiota is the key stage for its mechanism study. DHM is a flavonoid with poor oral bioavailability *in vivo* because of its rare absorption in the gastrointestinal tract (GI). Due to the low bioavailability, the vast majority of DHM persists in the colon where it is exposed to the gut microbiota, which markedly alters the richness and diversity of the gut microbiota and modulates the gut microbiota composition [88]. Previous studies indicated that DHM could be distributed widely in different organs such as the liver, kidney, lung, brain, and heart, whereas most of them were eliminated in feces, which indicated that DHM is predominantly distributed in the intestinal tract, and closely interacts with the gut microbiota [89].

It is reported that DHM treatment could obviously change the relative abundances of gut microbiota at different levels [90]. DHM is able to dramatically increase the abundance of *Bacteroidetes* but decrease the abundance of *Firmicutes*, which was related to obesity intervention in humans. In brief, decreasing the ratio of *Firmicutes* to *Bacteroidetes* was demonstrated to control body weight via modulatory glucose and lipid metabolism. Besides, DHM supplement can decrease the abundances of *Lachnoclostridium*, *Alistipes*, *Ruminococcaceae UCG-010*, *Allobaculum*, *Ruminiclostridium 9*, *Rikenellaceae RC9*, *Ruminococcaceae UCG-005*, *Anaerotruncus*, *Defluviitaleaceae UCG-011*, [*Eubacterium*] *ventriosum*, *Christensenellaceae R-7*, and *Odoribacter*, whereas it can increase the abundances of *Parasutterella*, *Erysipelatoclostridium*, and *Parabacteroides* [91]. Thus, a lot of evidences suggested that DHM supplement could intervene against chronic diseases, such as obesity, diabetes, and cancers, via modulating the gut microbiota composition [92].

Moreover, the interaction between DHM and gut microbiota is reported to be associated with cancer. DHM was reported to promote the CPT-11 effect both in the mouse model of AOM/DSS cancer; tumors were sensitive to 100 mg/kg DHM chemotherapy under 100 mg/kg or 200 mg/kg CPT-11 (irinotecan). DHM-driven CPT-11 chemotherapy induced enhanced IgG levels and the reduction of *Fusobacterium* abundance in the gut [91]. Besides, the intestinal tract is the most import target organ for DHM intervention associated with the chemotherapeutic efficacy and reduced risk of the side effects of cancer treatment via the regulation of immune responses and the shaping of gut microbiota [92].

On the other hand, DHM was reported to be biotransformed into other metabolites by gut microbiota *via* methylation, reduction, dehydroxylation, glucuronidation, and sulfation pathways which may be closely related to the regulation of redox balance [93]. Therefore, gut microbiota is a potential important interface for regulating the redox balance for providing more therapeutic schedules on various other human diseases.

## 6. Conclusions and Perspectives

**Figure 2:**
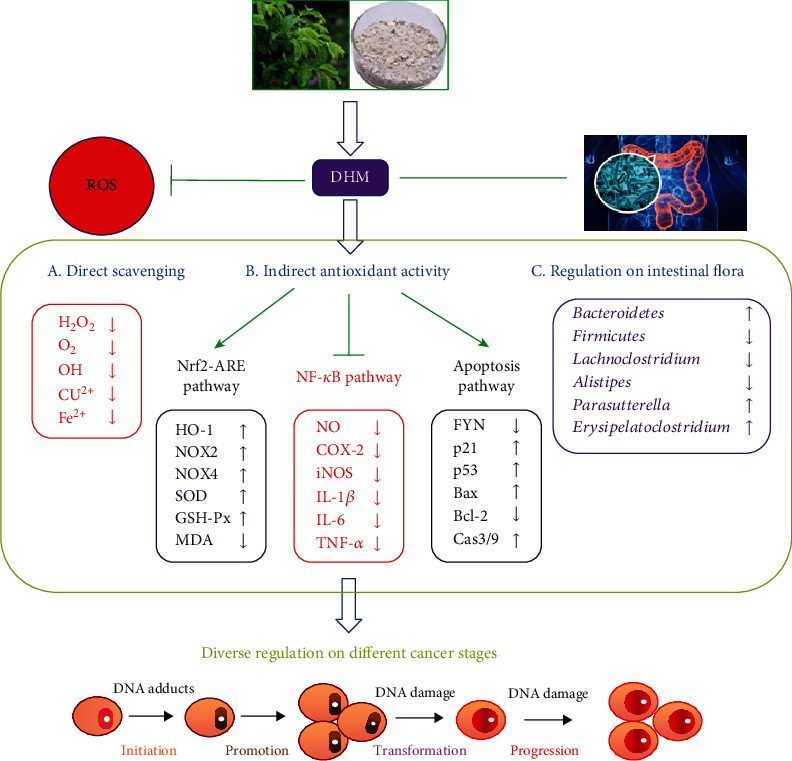
Molecular mechanism diagram of the inhibitory effect of DHM on cancer.

DHM originated from a natural plant in China and has received increasing attention due to its pharmacological activities. In this review, the physicochemical properties of DHM have been mentioned. Its antioxidant, anti-inflammatory, and anticancer activities and related molecular mechanisms have been further reviewed. The molecular mechanism diagram of the inhibitory effect of DHM on cancer has been summarized as shown in Figure 2. In brief, DHM exerts its anticancer activity via direct scavenging of ROS, regulating intestinal microbiota, and indirectly modulating the cellular signaling pathway, including the activation of the Nrf2-ARE pathway, the inhibition of the NF-*κ*B pathway, and the induction of the apoptosis pathway.

Substantial scientific evidence about the functional properties of DHM is available from cell culture and animal studies. DHM exhibited bioactivities by modulating several molecular pathways. However, the mechanism of pharmacological action, distribution, and metabolism are still not well investigated *in vivo* in animals, and what is more, the evidence from clinical studies is meager. The novel targets of the signaling transduction of DHM still require more work. Furthermore, more studies are needed in humans in order improve the applications of DHM in food and pharmaceutical industries. The future *in vivo* researches by multiomics technologies are required to understand the safety, bioavailability, and metabolism mechanism of DHM targeting on oxidative stress, inflammation, and cancer, especially to reveal the reciprocal interaction among DHM, cells/organs, and gut microbiota. This could pave ways for the industry applications of DHM as a functional food/healthy food/therapeutic agent.

## Figures and Tables

**Table 1:** The chemopreventive effect and potential mechanism of dihydromyricetin in cancer.

Origin	Cell lines/animals/human	Treatment methods	Mode of administration	Dose and duration time	Mechanism of action/activities/effects showed	Reference
DHM prepared from *A. grossedentat*a with a purity of 98%	Osteosarcoma cells	Cell cycle and apoptosis analysis		15, 30, and 60 *μ*M for 24 or 48 h	(1) Exhibited anticancer activity through increased p21 expression and G2-M cell cycle arrest, caused DNA damage, activated ATM-CHK2-H2AX signaling pathways, and induced apoptosis in osteosarcoma cells (2) Antitumor could be due to the activation of AMPK and p38MAPK pathways	Zhiqiang et al. (2014)
Ampelopsin ≥ 98%	Non-small-cell lung cancer (NSCLC) cells	Cell death analysis		20 *μ*M for 48-72 h	DHM in combination with erlotinib induced cell death via the NOX2-ROS-Bim pathway in NSCLC cells	Seung-Woo et al. (2017)
Ampelopsins A and C isolated from the roots of *V. thunbergii* with a purity of 98.5 and 99.0%, respectively	MDA-MB-231 breast cancer cells	Metastasis analysis		Ampelopsin A (10−50 *μ*M) and Ampelopsin C (1−5 *μ*M) for 24−72 h	Inhibited metastasis of MDA-MB-231 cells by downregulating the AxL, TYRO3, and FYN expressions	Cheng et al. (2019)
Ampelopsin ≥ 98%	MCF-7 and MDA-MB-231 breast cancer cells	Cell viability and increased apoptosis analysis		60 *μ*M for 24 h	DHM inhibited cell viability and increased apoptosis in MCF-7 and MDA-MB-231 breast cancer cells through ROS generation and ER stress pathway	Yong et al. (2014)
Ampelopsin (purity not provided)	HepG2 cells	Apoptosis analysis		0, 12.5, 25, 50, 100, 150, and 200 *μ*g/mL for 12, 24, and 36 h	(1) Induced apoptosis of HepG2 cells (2) Enhanced the levels of death receptor 4 (DR4) and death receptor 5 (DR5) and reduced the expression of Bcl-2 protein	Shimei et al. (2015)
Ampelopsin ≥ 98%	A549 human adenocarcinoma lung epithelial cells	Apoptosis analysis		0, 10, 20, or 30 *μ*M for 48 h	(1) Induced apoptosis in A549 cells (2) Reduced Bcl 2 and increased Bax levels (3) Cleaved PARP and reduced XIAP and survivin expression levels (4) Cleaved poly(ADP-ribose) polymerase expression	Xin-mei et al. (2015)
DHM ≥ 98%	Mouse hepatocellular carcinoma cells (Hepal-6)	Cell viability and apoptosis analysis		10, 50, or 100 *μ*M for 6 h, 12 h, and 24 h	DHM inhibited cell viability and induced apoptosis by downregulating ROS production via the TGF *β*/Smad3 signaling pathway in Hepal 6 cells	Bin et al. (2015)
AMP extracted from *A. megalophylla* (purity not provided)	HeLa cells	Apoptosis analysis		0, 30, 40, 50, 60, or 70 *μ*M for 8 h or 12 h	Induced apoptosis in HeLa cells through activation of caspases 9 and 3	Peipei et al. (2017)
DHM ≥ 98%	Hepatoma cell lines SK-Hep-1 and MHCC97L	Growth inhibition assays		0, 10, 50, and 100 *μ*mol/L for 24 h	DHM inhibited the migration and invasion of hepatoma cells via reducing the phosphorylation levels of p38, ERK1/2, and JNK	Qing-Yu et al. (2014)
DHM with a purity of 95%	HepG2 cells	Apoptosis analysis		0, 10, 20, and 30 *μ*M for 24 h	(1) Inhibition of the Akt/Bad signaling pathway (2) Upregulated the levels of mitochondrial proapoptotic proteins Bax and Bad (3) Inhibited the expression of the antiapoptotic protein Bcl-2 and enhanced the cleavage and activation of caspase 3 (4) Degradation of poly(ADP-ribose) polymerase	Zhuangwei et al. (2017)
DHM ≥ 98%	Human melanoma SK-MEL-28 cells	Cell cycle and apoptosis analysis		0, 50, and 100 *μ*M for 24, 48, or 72 h	(1) DHM inhibited cell proliferation (SK-MEL-28) cells through cell cycle arrest at the G1/S phase (2) Increased the production of p53 and p21 and downregulated the Cdc25A, Cdc2, and P-Cdc2 proteins (3) Induced apoptosis through enhancing the expression levels of Bax proteins and decreasing the protein levels of IKK-*α*, NF-*κ*B (p65), and P-p38	Guofang et al. (2014)
DHM ≥ 98%	AGS human gastric cancer cells	Cytotoxicity assays		25, 50, and 100 *μ*M for 48 or 72 h	DHM inhibited AGS cell proliferation and induced cell cytotoxicity through the regulation of expression of apoptotic genes such as p53 and B-cell lymphoma-2	Ji et al. (2015)
DHM ≥ 98%	HepG2 cells	Cell growth inhibition assays		5, 10, 25, and 50 *μ*M for 6, 12, 24, and 48 h	DHM-induced autophagy inhibited cell proliferation through suppressing the activation of mTOR and regulating upstream signaling pathways such as ERK1/2, AMPK, and PI3K/PDK1/Akt pathways	Juan et al. (2014)
DHM > 9%	Human choriocarcinoma cell line (JAr cells)	Apoptosis assays		0, 40, 60, and 100 mg/L for 48 h	Inhibited proliferation of JAr cells by inducing apoptosis through increasing protein expression level of BCL-2 associated X, and associated protein, and decreased the levels of BCL-2 and procaspase 3	Yanzhen et al. (2018)
DHM (purity not provided)	Human ovarian cancer A2780 and SKOV3 cells	Apoptosis assays		25, 50, and 100 *μ*M for 24 or 48 h	DHM inhibited the ovarian cancer cells and induced cell apoptosis through p53-mediated downregulation of survivin	Yingqi et al. (2017)
DHM ≥ 98%	HepG2, QGY7701, QGY7703, Huh 7, QSG7701, MHCC97L and H, and SK-Hep-1 cells	Cell proliferation and apoptosis assays		0, 50, and 100 *μ*M for 24, or 48 h	(1) DHM inhibited cell proliferation and induced cell apoptosis in hepatocellular carcinoma cells (2) Apoptosis was induced through upregulating p53 expression, and the upregulation of p53 increased the levels of cleaved-caspase 3 protein	Jie et al. (2014)
DHM ≥ 98%	SK-MEL-28 human melanoma cells	Apoptosis		25, 50, and, 100 *μ*M for 24 h	Enhanced cell death and apoptosis by regulating the NF-*κ*B signaling pathway	Ding-Zhou et al. (2017)
DHM ≥ 98%	Hepatocellular carcinoma cells	Apoptosis		25-200 *μ*M for 12 or 24 h	DHM with Nedaplatin (NDP) inhibited growth and induced apoptosis through the activation of the p53/Bcl-2 signaling pathways	Lianggui et al. (2015)
DHM ≥ 98%	A549 lung carcinoma cells and fibroblasts	Growth inhibition assays		0, 1, 5, and 10 *μ*M for 48 h	DHM inhibited the growth of fibroblasts in the lung cancer cells via the activation of Erk1/2 and Akt signaling pathways	Kai-jie et al. (2017)
DHM ≥ 98%	Hepatocellular cancer cells (HepG2 and Hep3B)	Growth inhibition and cell cycle assays		2, 10, 50, 100, and 200 *μ*M for 48 h	Inhibited proliferation of the cells via G2/M phase cell cycle arrest through the Chk1/Chk2/Cdc25C signaling pathway	Haili et al. (2013)
DHM > 99%	Human choriocarcinoma JAR cells	Growth inhibition and cell cycle assays		0, 40, 60, and 100 mg/L for 48 h	DHM inhibited the proliferation of JAR cells through cell cycle arrest via the downregulation of cyclin A1, cyclin D1, SMAD3, and SMAD4 expression levels	Yanzhen et al. (2020)
DHM > 98%	Human ovarian cancer SKOV3 cells	Cell migration, invasion, and apoptosis assays		80 and 120 *μ*M for 48 h	(1) Exhibited anticancer activity by reducing cell migration and invasion (2) Induced cell apoptosis via upregulation of cleaved-caspase 3 and the Bax/Bcl-2 ratio (3) Inhibited GRASP65 expression and the regulation of the JNK/ERK pathway	Fengjie et al. (2019)
DHM 98%	Human non-small-cell lung cancer (A549 and H1975) cell lines	Cytotoxic and apoptosis assays		0, 50, 75, and 100 *μ*M for 24 h	Exhibited cytotoxic effect by inducing apoptosis through Bcl-w suppression-mediated mitochondrial membrane depolarization, caspase 9/7/3 activation, and poly(ADP-ribose) polymerase (PARP) cleavage in A549 and H1975 cells	Shang-Jyh et al. (2017)
DHM (purity not provided)	Human gastric carcinoma cells (SGC7901 and SGC7901/5-FU)	Proliferation inhibition assays		1.25 and 2.5 *μ*g/mL for 48 h	Inhibited proliferation of both SGC7901 and SGC7901/5-FU cells through the downregulation of the MDR1 expression	Mingcai et al. (2020)
DHM ≥ 98%	Human hepatocarcinoma (HepG2) cells	Apoptosis assays		10, 50, and 100 *μ*M for 24 h	DHM-induced apoptosis of human hepatocellular carcinoma cells through a ROS-related pathway	Bin et al. (2014)
Ampelopsin (purity not provided)	Breast cancer MDA-MB-231 cells and rats	MNU-induced breast cancer in rats	Orally fed	10, 25, 50 *μ*M for 48 h and 50 and 100 mg/kg BW for 18 weeks	Inhibited the cancer cells effectively in vitro and in vivo through effectively suppressing mammalian target of rapamycin (mTOR) activity in breast cancer	Chang et al. (2014)
Ampelopsis isolated from *A. grossedentata* with 80%	PC-3 human prostate cancer cells and mice prostate cancer model	Cell migration, invasion, growth inhibition, and apoptosis assays	Oral gavage	0, 25, and 50 *μ*M for 48 h. 150 and 300 mg/kg BW for 8 weeks	(1) Inhibited the migration and invasion of PC-3 cells in vitro (2) Decreased the growth of PC-3 tumors and lymph node and lung metastases in a dose-dependent manner in mice (3) Exhibited anticancer activity via induction of apoptosis, reduction of prostate tumor angiogenesis, and reduction of CXCR4 expression	Feng et al. (2012)
Ampelopsin-sodium (purity not provided)	BALB/c mice	Mice implanted with human bladder carcinoma EJ cells and murine sarcoma 180 cells	IP/IV/II administration	160, 200, and 260 mg/kg BW for 2-3 weeks	DHM considerably inhibited the proliferation of EJ and sarcoma 180 cells both in vivo and in vitro	Baolai et al. (2012)
Ampelopsin ≥ 98%	Human glioma cell lines U251 and A172, and male BALB/c-nu mice xenograft model	Apoptosis and tumor growth inhibition	Intraperitoneal administration	25, 50, 100 *μ*M for 24 h and 50 and 100 mg/kg BW for 30 days	(1) Induced apoptosis by arresting at G1 and S phases and autophagy through potentiating ROS generation and JNK activation in human glioma cells (2) DHM activated caspase 8, caspase 9, and caspase 3 contributing to PARP cleavage (3) Reduced tumor growth of human glioma xenograft in mice	Zhigang et al. (2019)
DHM 98%	Colo-205 cells and xenograft tumor transplant mice	Cell growth inhibition assays	Intragastric administration	25, 50, and 100 mg/kg BW for 21 days	Inhibited the proliferation and growth of Colo-205 colon cancer cells considerably in vivo and in vitro via suppression of the expression and secretion of Sema4D	Jun et al. (2019)

**Table 2:** Antioxidant activities and mechanisms of the action of DHM.

Origin	In vitro/cell culture	Methods of antioxidant activities measure	Dose and duration time	Results	Reference
DHM ≥ 99.5%	RAW264.7 cells	Lipopolysaccharide- (LPS-) induced oxidative stress	DHM treated at 0 to 50 *μ*g/mL for 2 hours	DHM reduced LPS-induced oxidative stress through inhibiting the production of reactive oxygen species (ROS) and enhanced the antioxidant system by activating superoxide dismutase (SOD) and the Nrf2/HO-1 pathway	Xuejun et al. (2018)
DHM ≥ 98%	HepG2 cells	Reactive oxygen species (ROS)	DHM at 10, 50, or 100 mM for 6 h, 12 h, and 24 h	DHM reduced ROS accumulation in a concentration-dependent manner in HepG2 cells	Bin et al. (2014)
Ampelopsin purity 95%	In vitro assays	DPPH, ABTS, H_2_O_2_, and O_2_ radical methods	37°C for 15, 20, and 60 minutes	DHM inhibited free radicals. EC_50_ values of DHM for scavenging DPPH, ABTS, H_2_O_2_, and O_2_ radicals were 8.18, 5.32, 7.95, and 7.79 (*μ*g/mL), respectively	Xiang et al. (2017)
Ampelopsin 98%	PC12 cells	Reactive oxygen species	Ampelopsin at 50 and 100 *μ*M for 1 h	DHM inhibited reactive oxygen species in 6-OHDA stimulated PC12 cells in concentration-dependent manner	Xianjuan et al. (2015)
Ampelopsin ≥ 98%	Glomerular mesangial cells (MCs)	ROS and ROS enzymes	DHM at 0, 10, 20, and 40 *μ*M for 24 h	DHM inhibited the intracellular ROS production and expression levels of ROS-producing enzymes NADPH oxidase 2 (NOX2) and NOX4 and mediated the antioxidative effects through the activation of Nrf2/HO-1 pathway	Chunping et al. (2020)
Ampelopsin > 98%	In vitro assays	Hydroxyl and superoxide radical methods	Ampelopsin 10 to 100 *μ*M for 60 min and 25°C for 20 min	Ampelopsin eliminated ^•^OH and O_2_^•–^ in a concentration-dependent manner; the EC_50_ values were 29.4 ± 4.1 *μ*M and 88.9 ± 9.4 *μ*M	Jiantao et al. (2008)
Ampelopsin > 98%	PC12 cells	Reactive oxygen species	Ampelopsin at 1, 5, and 15 mg/mL for 1 h	Inhibited the formation of reactive oxygen species (ROS) and enhanced the cellular antioxidant defense through activation of the ERK and Akt signaling pathways in PC12 cells	Xianjuan et al. (2011)
DHM 64.7%	In vitro assay	DPPH	DHM at 2, 4, 6, 8, 10, and 12 ppm for 30 min	DHM extract inhibited DPPH radicals with IC_50_ value of 3.9 ppm	Liyun et al. (2015)
DHM ≥ 98%	In vitro assays	DPPH and ORAC	DHM at 12.5, 25, 50, 100, 200, and 400 *μ*g/mL for 30 min	DHM dose-dependently inhibited the DPPH and ORAC radicals	Kun et al. (2019)
DHM ≥ 98%	HepG2 cells	Nrf2/Keap1 pathway	40 *μ*M for 3-12 h	Exhibited antioxidant activity by activating the cellular Nrf2/Keap1 pathway	Kun et al. (2019)
Not provided	(HEI-OC) 1 auditory cells	ROS	DHM at 10, 100, and 1000 *μ*M for 24 h	DHM inhibited ROS accumulation in HEI-OC cells	Hezhou et al. (2020)
DHM > 98%	B16F10 mouse melanoma cells	Reactive oxygen species	1, 25, and 50 *μ*M for 24 h	DHM reduced intracellular reactive oxygen species and reactive species (RS) levels	Huey-Chun et al. (2016)
DHM > 98%	Rat cardiac fibroblasts	Ang II-induced oxidative stress	0–320 *μ*M for 4 h or 80 *μ*M for 0–24 h	DHM inhibited cellular reactive oxygen species production and MDA level, and enhanced the SOD activity and total antioxidant capacity (T-AOC)	Qiuyi et al. (2017)
DHM ≥ 98%	In vitro assays	DPPH and ABTS	100 mg/mL for 6 min or 30 min	Cookies fortified with DHM significantly enhanced the DPPH and ABTS radical scavenging activities	Jing et al. (2018)
DHM 97%	PC12 cells	Methylglyoxal- (MG-) induced oxidative stress in PC12 cells	20 and 10 mol/L for 24 h	Inhibited the intracellular ROS and modulating AMPK/GLUT4 signaling pathway in PC12 cells	Baoping et al. (2014)
DHM 95%	In vitro assay	DPPH	0 to 50 *μ*g/mL for 30 min	DHM and lecithin complex inhibited DPPH radicals with IC_50_ value of 22.60 *μ*g/mL	Benguo et al. (2009)
DHM > 98%	Rat cardiomyocytes	Ang II-stimulated reactive oxygen species in cardiomyocytes	20, 40, 80, and 160 *μ*M for 8 h, 12 h, 24 h, or 48 h	DHM reduced ROS generation in Ang II-stimulated cardiomyocytes by increasing total antioxidative capacity through upregulating expression of SOD and thioredoxin	Guoliang et al. (2015)
DHM (purity not provided)	HUVECs	Sodium nitroprusside- (SNP-) induced oxidative damage	300 *μ*mol/L for 2 hours	DHM reduced ROS overproduction, decreased MDA level and increased SOD activity and showed antioxidant activity in HUVECs by activating the PI3K/Akt/FoxO3a signaling pathway	Xiaoying et al. (2019)
DHM 99%	Human umbilical vein endothelial cells (HUVECs)	H_2_O_2_-induced oxidative stress	37.5-300 *μ*M for 2 h	DHM inhibited intracellular ROS overproduction in HUVECs cells	Xiaolong et al. (2015)
Ampelopsin ≥ 95%	PK-15 cells	H_2_O_2_-induced oxidative stress in PK-15	0, 15, 30, and 60 *μ*g/mL for 1 h	Significantly decreased MDA production in H_2_O_2_-induced PK-15 cells	Tan et al. (2010)
DHM 98%	Colo-205 cells	Reactive oxygen species and MDA	8, 16, and 32 *μ*M for 2 h	Inhibited reactive oxygen species and malondialdehyde levels	Jun et al. (2019)
Ampelopsin 98%	In vitro assay	DPPH method	0.1 to 0.4 *μ*g/mL for 60 min	DHM inhibited DPPH radicals with IC_50_ value of 0.235 *μ*g/mL	Wenzhen et al. (2014)
DHM 98%	In vitro assays	DPPH, ABTS, O_2_ radical, and Fe^2+^ chelating method	2 to 20 *μ*g/mL for 30 min	Eliminated ABTS, DPPH free radicals, reduced Cu^2+^, and chelated Fe^2+^	Xican et al. (2016)
DHM ≥ 98%	C57BL/6J mice	Streptozotocin-induced oxidative stress model	DHM at 100 mg/kg/day for 14 weeks	DHM decreased MDA and increased the SOD and GSH-Px	Bin et al. (2017a)
DHM (purity not provided)	Male C57BL/6 mice	Transverse aortic constriction induced oxidative stress in mice	DHM (250 mg/kg/day) for 2 weeks	DHM administration reduced reactive oxygen species and malondialdehyde level, and increased total antioxidant capacity and SOD activity in mice	Yun et al. (2018)
DHM > 98.0%	Chickens	LPS-induced oxidative stress in chickens	0.025%, 0.05%, and 0.1% for 14 days	DHM increased SOD and GSH-Px activity and GSH in chicken plasma and ileum	Yicong et al. (2020)
DHM (purity not provided)	ICR mice	Sleep deprivation induced oxidative stress	100, 50, and 25 mg/kg/day for 14 days	DHM increased SOD activity and reduced MDA level	Hongxiang et al. (2019)
DHM (purity not provided)	Rat	DPPH radical scavenging activity of rat serum	100 mg/kg/BW	DHM increased the antioxidative capacity of rat serum against DPPH radicals	Xiao et al. (2014)
Ampelopsin 95%	Piglets	LPS-induced oxidative stress in piglets	2.5, 5, and 10 *μ*g/mL for 30 min	Decreased the MDA and protein carbonyl levels in LPS-treated piglets	Xiang et al. (2014)

**Table 3:** Anti-inflammatory activities and mechanisms of the action of DHM.

Treatment methods	Mode of administration	Dose and duration time	Mechanism of action/activities/effects showed	Reference
LPS-induced inflammation		50, 100, and 150 *μ*g/mL for 2 h	Showed anti-inflammatory activity by inhibiting interconnected ROS/Akt/IKK/NF-*κ*B signaling pathways. Significantly inhibited the release of nitric oxide (NO) and proinflammatory cytokines such as IL-1*β*, IL-6, and TNF-*α* in a dose-dependent manner	Shimei et al. (2012)
LPS-induced inflammation in RAW264.7 cells		0.4, 0.8,1.5, 3, 6.2, 12.5, 25, 50, and 100 *μ*M for 24 h	Showed anti-inflammatory effects through the inhibition of the release of nitric oxide (NO) in RAW macrophages	Yuemei et al. (2019)
A549 cells were stimulated with TLR3 agonist poly(I : C)		25, 50, and 100 *μ*M for 3 days	Attenuated inflammation through TLR3 pathway	Yuanxin et al. (2020)
TNF-*α*-induced inflammation in HUVECs		5, 10, 25, 50, 75, and 100 𝜇M for 24 h	Attenuated endothelial dysfunction induced by TNF-*α* in a miR-21-dependent manner	Dafeng et al. (2018)
TNF-*α*-induced inflammation		50–200 *μ*M for 24 hours	Showed anti-inflammatory activity via suppression of TNF-*α*-induced NF-*κ*B activation	Nina et al. (2016)
LPS-induced cardiomyocyte inflammation		25, 50, and 100 *μ*M for 12 h	Exhibited anti-inflammatory activity in cardiomyocytes by reducing TNF-*α* and IL-6 levels via inhibition of the TLR4/NF-*κ*B signaling pathways	Meng-qiao et al. (2017)
LPS-induced inflammation in BV-2 cells		10, 25, and 50 *μ*M for 24 h	DHM significantly reduced LPS-induced NO, IL-6, and TNF-*α* production and levels of iNOS and COX-2 in BV-2 cells	Yafei et al. (2017)
LPS-induced inflammation in BV-2 cells		20, 40, 80, or 100 mg/L for 48 h	DHM exhibited the anti-inflammatory effect on LPS-induced BV-2 microglial cells through the TRL4/NF-*κ*B signaling pathway and suppressed the levels of IL-6, IL-1*β*, and TNF-*α*, and inhibited the protein expression of iNOS and COX-2	Nianshui et al. (2019)
TPA-induced acute inflammation/LPS-induced RAW 264.7 macrophage cells	Topical application	2.3 and 4.6 mg per ear for 5 h/37.5, 75, 150, and 300 *μ*M for 2 h	Showed anti-inflammatory activity through suppressing the activation of NF-*κ*B and the phosphorylation of p38 and JNK. Inhibited the levels of proinflammatory cytokines such as TNF-*α*, IL-1*β*, and IL-6 as well as increased the level of the anti-inflammatory cytokine IL-10 in LPS-treated mice. Reduced the protein expression of iNOS, TNF-*α*, and COX-2 in RAW cells	Hou et al. (2015)
LPS-induced inflammation	Amp was dissolved in dimethylsulfoxide (DMSO), and dilutions were made in DMEM	0.5 *μ*g/mL for 24 h	Reduced the phosphorylation levels of JAK2-STAT3 and STAT3 nuclear translocation and suppressed LPS-induced activation of the I*κ*B/NF-*κ*B inflammation pathway. Decreased the production of NO and PGE2 and suppressed the expression of iNOS and COX-2 and reduced proinflammatory cytokines such as IL-1*β*, IL-6, and TNF-*α*	Leihua et al. (2017)
Ovalbumin- (OVA-) induced inflammation in C57BL/6 mouse	Administered intraperitoneally	10 mg/kg BW for 14 days	DHM exhibited anti-inflammatory activity by reducing the levels of IL-4, IL-5, and IL-13 in the bronchoalveolar lavage fluid in an OVA-induced asthma model	Bin et al. (2017)
Doxorubicin- (DOX-) induced cardiotoxicity rat model and DOX-induced H9C2 cells	Administered intragastrically	100 mg/kg/day or 200 mg/kg/day for 6 weeks	DHM protected against DOX-induced cardiotoxicity by inhibiting NLRP3 inflammasome activation via stimulation of the SIRT1 pathway and suppressed IL-1*β* and IL-18 release, and upregulated SIRT1 protein levels in vivo and in vitro	Zhenzhu et al. (2020)
Cecal ligation and puncture- (CLP-) induced lung injury model	Orally administered	50, 100, 150 mg/kg/day for 3 days	DHM treatment significantly inhibited the CLP-induced NLRP3 inflammasome pathway, IL-1*β*, and IL-18	Yu-Chang et al. (2019)
LPS-mediated inflammation	Diet supplemented with ampelopsin	100 and 400 mg/kg BW for 28 days	Showed the anti-inflammatory activity through the reduction of activation of AKT and STAT3 phosphorylation and suppressed the DNA-binding activity of NF-*κ*B. Decreased the proinflammatory mediators such as TNF-*α*, IL-1*β*, IL-6, and COX-2	Xiang et al. (2017)
Double-blind clinical trial	Orally administered	Four 150 mg capsules daily for 12 weeks	Exhibited anti-inflammatory activity by decreasing the serum levels of TNF-*α*, cytokeratin-18 fragment, and fibroblast growth factor 21	Shihui et al. (2015)
APP/PS1 double transgenic mice	Injected intraperitoneally	1 mg/kg BW for 2 and 4 weeks	DHM reduced activation of NLRP3 inflammasomes and reduced expression of NLRP3 inflammasome components and decreased IL-1*β* in transgenic mice	Jie et al. (2018)
Collagen-induced inflammation	Intraperitoneally	5, 25, and 50 mg/kg BW every other day for 5 weeks	Alleviated inflammation in rats by attenuating IL-1*β* production via suppression of NF-*κ*B signaling	Jing et al. (2019)
Lead- (Pb-) stimulated inflammation	Oral gavage administration	125 and 250 mg/kg/BW for 3 months	Inhibited Pb-induced inflammation by regulating the AMPK, A*β*, TLR4, MyD88, p38, and GSK-3*β* pathways	Chan-Min et al. (2018)
Caerulin-induced inflammation in mice and BMDMs	Intraperitoneally	Single dose of 25/100 mg/kg	Inhibited production of proinflammatory cytokines IL-1*β*, TNF-*α*, and IL-17 in mice and BMDMs	Rongrong et al. (2018)
LPS-induced inflammation in chickens	Feeding in the diet	0.025, 0.05, and 0.1 mg/kg for 14 days	DHM reduced inflammation by inhibiting NLRP3 inflammasome and TLR4/NF-*κ*B signaling pathway in ileum in chickens	Yicong et al. (2020)
Mice transplanted with Colo-205 cells	Intragastric administration	25, 50, and 100 mg/kg for 21 days	Decreased the levels of IL-1*β*, IL-6, IL-8, and TNF-*α* as well as reduced the expression of COX-2 and iNOS	Jun et al. 2019
LPS-induced inflammation in lung tissue	Oral gavage administration	500 mg/kg BW for 4 days	Exhibited anti-inflammatory effects by inhibiting the MAPK signaling pathway as well as TNF-*α*, IL-1𝛽, and IL-6 levels	Bo et al. (2018)
A rat model of inflammation induced by collagen	Intraperitoneally	5, 25, and 50 mg/kg for 5 weeks	Exhibited anti-inflammatory effects by inhibiting NF-*κ*B signaling pathway	Jing et al. (2020)
Carrageenan-induced paw edema in rat and LPS-induced inflammation in RAW264.7 model	Intraperitoneal injection	50, 100, and 250 mg/kg for 7 days	DHM significantly reduced rat paw edema induced by carrageenan and noticeably inhibited NO secretion, iNOS, and COX-2 protein expression and decreased p65 phosphorylation via suppression of IKK-*β* activity and IKK-*α*/*β* phosphorylation	Rui et al. (2016)
Complete Freund's Adjuvant- (CFA-) induced inflammation in rheumatoid arthritis model	Gavage administration	20 and 50 mg/kg per day for 25 days	DHM inhibited the expressions of proinflammatory cytokines IL-1*β*, IL-6, TNF-*α*, and COX-2 via activating the Nrf2 pathway	Jianguo et al. (2018)
Streptozotocin-induced diabetic inflammation	Intragastrically given	100 mg/kg/day for 14 weeks	DHM reduced the levels of proinflammatory factors such as IL-6 and TNF-*α*	Bin et al. (2017a)
Alcohol-induced inflammation in C57BL/6 mice	Incorporated in the diet	75 and 150 mg/kg BW for 6 weeks	DHM considerably alleviated the hepatic IL-1*β* and IL-6 levels	Ping et al. (2017)

## Abbreviations

AAPH: 2,2′-Azobis (2-amidinopropane) dihydrochloride
ABTS: 3-Ethylbenzothiazoline-6-sulphonic acid
BAD: Bcl-2-associated death promoter
BAX: BCL-2-associated X protein
COX-2: Cyclooxygenase 2
DHM: Dihydromyricetin
DPPH: 1,1-Diphenyl-2-picrylhydrazyl radical 2,2-diphenyl-1-(2,4,6-trinitrophenyl) hydrazyl
GSH: Glutathione
H_2_O_2_: Hydrogen peroxide
HCC: Hepatocellular carcinoma
HUVECs: Human umbilical vein endothelial cells
iNOS: Inducible nitric oxide synthase
IL: Interleukin
IC_50_: Half-maximal inhibitory concentration
LPO: Lipid peroxidation
LPS: Lipopolysaccharide
MDA: Malondialdehyde
MIC: Minimum inhibitory concentration
MAPKs: Mitogen-activated protein kinases
MNU: 1-Methyl-1-nitrosourea
NF-*κ*B: Nuclear factor-*κ*-gene binding
Nrf2: Nuclear factor erythroid 2-related factor 2
O_2_^•−^: Superoxide anion
ORAC: Oxygen radical absorption capacity
OH^•^: Hydroxyl radicals
ROS: Reactive oxygen species
SOD: Superoxide dismutase
STAT3: Transduction and transcription 3
TNF-*α*: Tumor necrosis factor-alpha
TLRs: Toll-like receptors.
